# Carborane-Containing Aromatic Polyimide Films with Ultrahigh Thermo-Oxidative Stability

**DOI:** 10.3390/polym11121930

**Published:** 2019-11-22

**Authors:** Fulin Liu, Guangqiang Fang, Haixia Yang, Shiyong Yang, Xuezhong Zhang, Zhijie Zhang

**Affiliations:** 1 Laboratory of Advanced Polymer Materials, Institute of Chemistry, Chinese Academy of Sciences, Beijing 100190, China; liufulin@iccas.ac.cn (F.L.);; 2 School of Chemical Sciences, University of Chinese Academy of Sciences, Beijing 100049, China; 3 Shanghai Institute of Aerospace Systems Engineering, Shanghai 201108, China

**Keywords:** polyimide, carborane, thermo-oxidative stability, self-healing layer

## Abstract

Carborane-containing aromatic polyimide (CPI) films with ultrahigh thermo-oxidative stability at 700 °C have been prepared by casting poly(amic acid) (PAA) resin solution on a glass surface, followed by thermal imidization at elevated temperatures. The PAA solution was prepared by copolymerization of an aromatic dianhydride and an aromatic diamine mixture, including carborane-containing aromatic diamine in an aprotic solvent. The CPI films showed excellent thermo-oxidative stability at 700 °C due to the multilayered protection layers formed on the film surface by thermal conversion of the carborane group into boron oxides. The boron oxide layer enhanced the degradation activation energy and suppressed the direct contact of inner polymer materials with oxygen molecules in a high-temperature environment, acting as a “self-healing” skin layer on the polyimide materials. The CPI-50 film was still flexible and maintained 50% retention of mechanical strength even after thermo-oxidative aging at 700 °C/5 min. The mechanism of thermo-oxidative degradation was proposed.

## 1. Introduction

Aromatic polyimide films have been extensively utilized in a variety of applications from microelectronic and optoelectronic devices to aerospace industries, owing to its excellent thermal stability, dielectric and electrical insulating performance, mechanical properties, and chemical and radiation resistance [1,2,3,4,5,6,7]. With the rapid development of high-tech fields, polyimide films have also been attempted to be used as gas separation membranes [8,9,10,11], battery separators [12,13,14,15], thermally conductive films [16,17,18], transparent electrodes [19,20,21,22], etc. Although polyimide is one of the most thermal stable polymers, it starts rapid thermal or thermo-oxidative decomposition at the beginning from 500–550 °C and loses most of its original weight at about 600–650 °C, which restricts its application in extreme environments, such as engine culverts in airplanes, nuclear power stations, and submarines. In those circumstances, high temperatures and repeated thermal cycles require thermal and thermos-oxidative stability of materials at elevated temperatures. In order to improve the thermal or thermo-oxidative stability, polyimide films are usually coated with metal or inorganic oxide protection layer or blended with inorganic fillers [23,24,25,26,27]. However, the mismatching of the coefficient of thermal expansion (CTE) between inorganic coatings and polymer matrices might result in serious troubles such as delamination, curl, or crack after harsh heat shocking [28]. Although the addition of inorganic fillers into polymer matrices to form hybrid structures is an effective pathway in enhancing thermo-oxidative stability of polymer materials, the limited filler loadings and the poor miscibility of inorganic fillers and organic matrices are the big issues [26,27,29,30]. Hence, developing intrinsic thermo-oxidative stable polymers is still the desirable pathway.

Incorporating the carborane (C_2_B_10_H_12_) cage structure into the polymer backbone has been found to provide a promising pathway to improve the thermo-oxidative stability of polymer materials due to the reaction of carborane with oxygen atoms to yield a boron oxide layer [31]. The icosahedral carborane structure has two carbon atoms in a symmetrical polyhedral cage, exhibiting three-dimensional aromaticity [32,33,34]. Y.P. Bai et al. synthesized wholly carborane-containing polyesters exhibiting a char yield of 64% in air and 47% in N2 at 700 °C, respectively, much superior to the carborane-free polyesters [35]. Zh. J. Zhang et al. synthesized a series of exactly alternating carborane-siloxane polymers showing excellent thermal stability with char yields of ≥83% both in air at 800 °C and in nitrogen at 1000 °C, respectively [36]. X. D. Jia et al. introduced a meta-carborane structure into the polybenzoxazine main-chain via click reaction showing a 5% weight loss at temperatures above 400 °C and high char yield of 76% in air at 800 °C [37].

In this paper, a series of carborane-containing aromatic polyimide (CPI) films with carborane cage structures in the polymer backbone have been prepared by copolymerization of aromatic dianhydride and a mixture of aromatic diamines, including 1,7-bis(aminophenyl)-*meta*-carborane. Experimental results indicated that the carborane containing polyimide films demonstrated great thermo-oxidative stability at temperatures of as high as 700 °C. The effect of polyimide structures on the thermal properties have been systematically investigated, and a mechanism of thermo-oxidative degradation was proposed. Combined with the neutron-trapping ability of the boron atoms, the thermal-oxidative stable CPI we reported were potential candidates in nuclear power applications.

## 2. Materials and Methods

### 2.1. Materials

3,3′,4,4′-biphenyltetracarboxylic dianhydride (*s*-BPDA), 4,4′-diaminodiphenyl ether (4,4′-ODA), and 1,4-benzenediamine (PDA) were obtained from Changzhou Sunlight Pharmaceutical Co., Ltd. (Changzhou, China), and dried in a vacuum at 160 °C, 80 °C, 80 °C for 12 h before use, respectively. The 1,7-bis(aminophenyl)-*meta*-carborane (BACB) was synthesized in our lab, and the details are shown in Scheme S1 in Supporting Information. *N*-methyl-2-pyrrolidinone (NMP) was purchased from J&K Scientific Co. Ltd. (Beijing, China), then distilled under reduced pressure with CaH_2_ and stored with 4Å molecular sieves before use. Other solvents and reagents were directly used without further purification.

### 2.2. Synthesis of the Carborane-Containing Polyimide Films

According to previous research, n(PDA)/n(4,4′-ODA) = 7/3 was the optimal molar ratio for thermal stability and mechanical property of the PI film to be in equilibrium [38]. Thus, the molar ratio of PDA and 4,4′-ODA was kept at 7/3 for all PI systems in this research. A typical polymerization process of CPI-20 was prepared in the following procedure: in a dried three-necked flask, equipped with an electric agitation, nitrogen gas inlet and outlet, PDA (1.8168 g, 16.8 mmoL), 4,4′-ODA (1.4417 g, 7.2 mmoL), BACB (1.9586 g, 6 mmoL), and part of anhydrous NMP (32 mL) were added. The mixture was stirred at room temperature until the diamine was completely dissolved, then an equimolar amount of dianhydride *s*-BPDA (8.8266 g, 30 mmoL) and residual NMP (10 mL) were added into the solution to adjust the solid content to approximately 25 wt.%. The mixture was continuously stirred at room temperature in N_2_ atmosphere for a further 48 h to get a homogeneous poly(amic acid) (PAA) solution. Then the viscous PAA solution was filtrated by G1 sand cored funnel and degassed under vacuum for 2 h in order to prepare a defect-free film. Subsequently, the PAA solution was coated onto a pre-cleaned glass plate and thermally imidized according to the following schedule: 120 °C/2 h, 200 °C/1 h, 300 °C/1 h, 400 °C/0.5 h with a heating rate of 2 °C min^−1^ to prepare the self-supporting films. As a consequence, reddish-brown CPI films with a thickness of about 50 µm were obtained. This series of CPI films were named as CPI-*x*, in which *x* represents the molar ratio of BACB. As a comparison, CPI-0 was based on *s*-BPDA, 4,4′-ODA, and PDA without any BACB, and its preparation and imidization processes were the same as above.

### 2.3. Characterization and Measurements

FT-IR spectra were measured in the range of 400–4000 cm^−1^ on a Tensor-27 Fourier transform spectrophotometer (Bruker, Germany). ^1^H-NMR spectra of PAA resins were acquired on a 400 MHz Avance 400 spectrometer (Bruker, Germany) in deuterated dimethyl sulfoxide (DMSO-*d*_6_). The elemental analysis of PAA resins was carried out with a Varlo ELIII elementar (Elementar, Langenselbold, Germany). The mechanical properties were conducted on an Instron 5567 universal testing machine (Instron, Norwood, MA, USA) with a specimen size of 80 × 10 × 0.05 mm^3^ in agreement with GB1447-2005 at a drawing rate of 2.0 mm min^−1^. Dynamic mechanical analyses were performed on a DMA Q800 thermal analysis system (TA, USA) with a heating rate of 5 °C min^−1^ under nitrogen atmosphere at a flow rate of 40 cm^3^ min^−1^. Thermogravimetric analyses were recorded on a TGA Q50 thermal analysis system (TA, USA) with a heating rate of 20 °C min^−1^ under both nitrogen and air atmosphere at a flow rate of 40 cm^3^ min^−1^. Thermo-oxidative aging was also carried out on a TGA Q50 thermogravimetric analyzer with a heating rate of 50 °C min^−1^ ramping from 50 to 700 °C and isothermal aging in air at 700 °C for 150 min. Surface chemistry of CPI samples and PAA resins were investigated by X-ray photoelectron spectroscopy (XPS, VG ESCALab 250Xi, USA) using 200 W monochromatic Al Kα radiation. Surfaces morphologies of the PI films were imaged by scanning electron microscopy (SEM, Hitachi S-4800, Tokyo, Japan) at 15 kV voltage.

## 3. Results and Discussions

### 3.1. Preparation and Characterization of CPI Films

**Figure 1:**
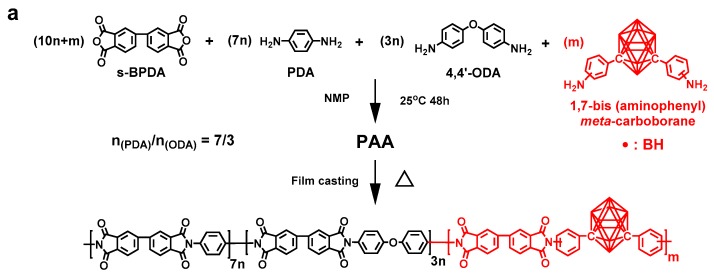

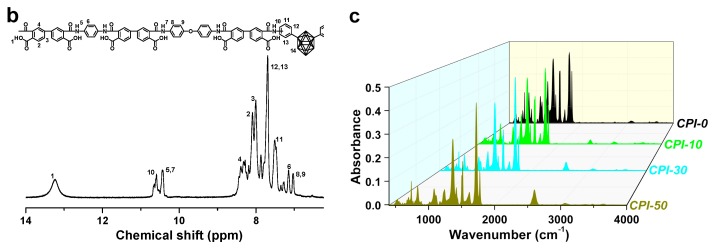
(**a**) Synthesis of the carborane-containing aromatic polyimide (CPI) films; (**b**) ^1^H-NMR spectrum of a representative poly(amic acid) (PAA) resin (CPI-50) and (**c**) FT-IR spectra of the CPI films.

The polyimide precursor resin—PAA resin solution was synthesized by polycondensation of equimolar aromatic dianhydride (*s*-BPDA) with a mixture of three different aromatic diamines, including PDA, 4,4′-ODA, and 1,7-bis(aminophenyl)-*meta*-carborane (BACB) in NMP (Figure 1a). The PAA solution was first coated on glass surface, followed by prebaking at 120 °C/2 h to remove part of the solvent, then thermally imidized in a vacuum at 200 °C/1 h, 300 °C/1 h, and 400 °C/0.5 h, respectively, to give a self-supporting CPI film with thickness of around 50 µm. A series of CPI-*x* films with various boron-loadings (*x* represents the molar ratio of BACB in diamine monomers and is equal to 0, 5, 10, 20, 30, 40, and 50, respectively) were obtained.

The PAA solution of CPI was trickled into excess ethanol to form a reddish-brown precipitate, which was immersed in ethanol for 48 h and dried under vacuum at 180 °C for 12 h to eliminate the NMP. Afterward, the dried PAA resin of CPI was characterized by ^1^H-NMR, XPS, and elemental analysis. Figure 1b shows ^1^H-NMR of a representative PAA resin (PAA-50), which was used for the preparation of CPI-50 film. The distinct splitting patterns were in well accordance with the expected PAA-50 chemical structure. Additionally, Table 1 illustrates the XPS and elemental analysis results of the PAA resin (PAA-50); although the XPS and elemental analysis could not detect specific elements, the contents of other elements were in great agreement with the calculated values. Furthermore, the chemical structures were also characterized by FT-IR spectroscopic measurements (Figure 1c). The intensity of the B-H characteristic absorptions around 2600 cm^−1^ became stronger and stronger with the increasing of boron loadings. The strong absorptions around 1775 and 1720 cm^−1^ referred to the asymmetric and symmetric stretching vibrations of C=O bands of imide rings in the polymer backbone. Other absorption bands collected near 1366 cm^−1^ and 3070 cm^−1^ were assigned to C–N and C_arom_–H, respectively. No signals around 3300~3500 cm^−1^ were detected in the FT-IR spectra, which meant neither –NH_2_ of BACB nor –CONH– of PAA existed in CPI films. That is, all the BACB was reacted with dianhydride, and the CPI film was completely imidized.

**Figure 2:**
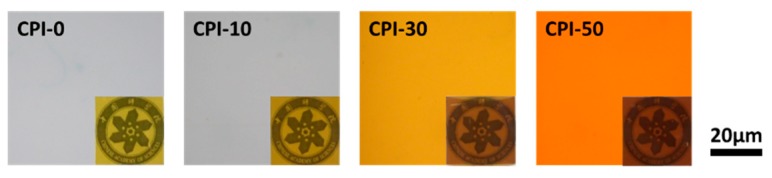
Polarizing microscopic images of the CPI films (the inserted images were the films covered on the logo of the Chinese Academy of Sciences).

Furthermore, no phase separation was detected by the polarizing microscopic images in all of the CPI films (Figure 2). The CPI films showed homogeneous and transparent images under polarized light, demonstrating that the carborane cage structures were linked into the polymer backbone instead of simply being dispersed as an inorganic filler. Since BACB is a characteristic reddish-brown powder, the color of the PAA solution deepened with increasing carborane loadings, as shown in Figure S1. Therefore, the color of the resultant CPI film deepened with increasing carborane loadings.

### 3.2. Thermal and Thermo-Oxidative Stabilities

**Figure 3:**
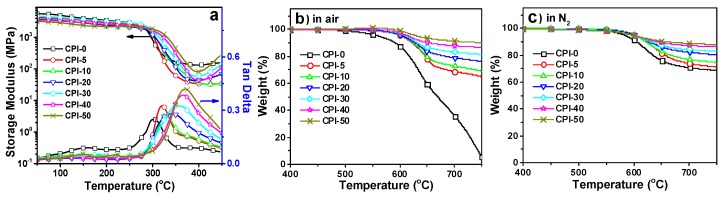
Thermal and thermo-oxidative stabilities of the CPI films; (**a**) DMA curves; (**b**) TGA curves in air; (**c**) TGA curves in N_2_.

Thermal stability characterized by DMA and TGA were compared in Table S1. The *T_g_* values (tan δ in DMA) increased from 304 °C (CPI-0) to 371 °C (CPI-50) with increasing boron loadings (Figure 3a). Although the bulky carborane cage structure enlarged the distance of polymer chain to chain, it also contrarily restricted the mobility of polymer chains in large-scale due to its big steric hindrance, and the movements induced by larger inter-chain space were too short in size scale to change polymers from a glassy state to rubbery state resulting in increased *T_g_* values.

Compared with the boron-free film (CPI-0), incorporating carborane into the polyimide backbone structure greatly enhanced polymer thermal and thermo-oxidative stabilities (Figure 3b,c). Both thermal and thermo-oxidative stability were improved with increasing boron-loadings. The initial thermal decomposition temperature (<1.0 wt.% of the original weight) were detected over 550 °C in air, 50 °C higher than that of CPI-0. The *T*_5_ in N_2_ were measured in the range of 595–601 °C, 13–19 °C higher than CPI-0, and apparently did not change with the boron-loadings. However, the *T*_5_ in air increased from 602 °C (CPI-5) to 632 °C (CPI-50) with increasing boron-loadings. At 50 mol.% of carborane loading (CPI-50), the *T*_10_ was measured at 657 °C (in N_2_) and 749 °C (in air), respectively, and the char yield at 750 °C was as high as 87.9% (in N_2_) and 90.0%(in air), indicating obvious improvement in thermo-oxidative stability. Moreover, the TGA analysis manifested that some weight gains were observed for the CPI films beginning at about 500 °C in air, which was probably attributed to the oxidation of boron atoms in the carborane cage to produce a boron oxide layer on the film surface, which could retard the further erosion of inner polymers [39].

### 3.3. Mechanical Properties

**Figure 4:**
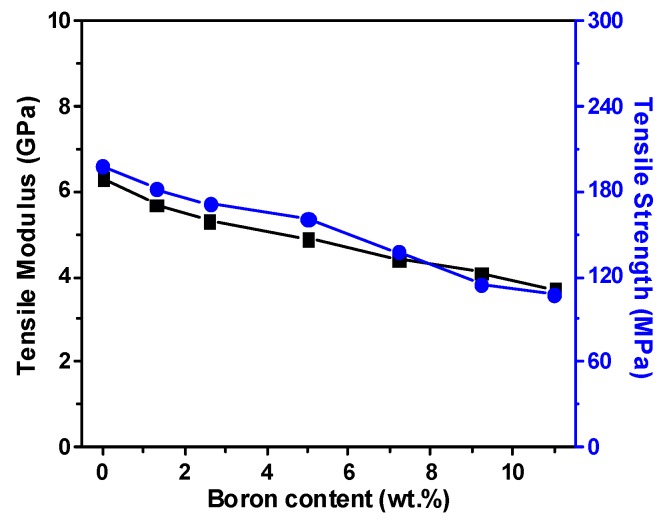
Tensile modulus and tensile strength of the CPI films vs. boron loadings.

During copolymerization with aromatic dianhydride, BACB is less reactive than the other two aromatic diamines (4,4′-ODA and PDA) due to the strong electron-withdrawing ability and the large steric hindrance effect of the carborane cage structure, diminishing the polymerization reactivity of the diamine monomer. Although it was hard for BACB to form high *M_w_* polymer, the BACB still reacted with dianhydride to form a lower *M_w_* polymer. As a consequence, the molecular weight of PAA resins was reduced, and the polymer inter-chain interaction was weakened with increasing boron-loadings, leading to a decreased tensile strength and modulus (Figure 4). Thus, the CPI-50 film showed the lowest mechanical performance with a tensile strength (*T_S_*) of 108 MPa and tensile modulus (*T_M_*) of 3.7 GPa, respectively, compared with CPI-20 (*T_S_*: 161 MPa, *T_M_*: 4.9 GPa).

**Figure 5:**
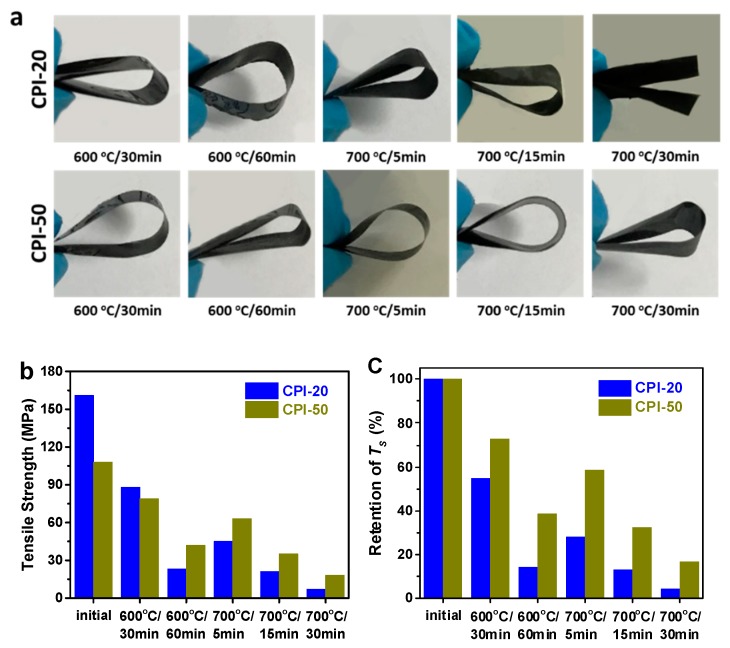
(**a**) The effects of bend testing; (**b**) tensile strength, and (**c**) retention of tensile strength of the CPI film samples after thermo-oxidative aging at various conditions.

CPI films with different boron-loadings (CPI-0, CPI-20, and CPI-50) were thermally treated in a muffle furnace under various thermo-oxidative aging conditions (600 °C/30 min, 600 °C/60 min, 700 °C/5 min, 700 °C/15 min, 700 °C/30 min, etc.). The flexibility of the CPI films was estimated by bend testing, and the tensile properties were measured on an Instron 5567 universal testing machine with a drawing rate of 2.0 mm min^−1^. Table 2 summarized the results of bend testing, after thermo-oxidative aging at 600 °C for 30 to 60 min, the boron-free films (CPI-0) were damaged into ashes or volatiles due to the violent degradation, and could not be tested. However, all the boron-containing films could be bent 180° under the same condition, showing great retention of the flexibility (Figure 5a). Additionally, after thermo-oxidative aging at 700 °C/30 min, although the CPI-20 film was broken into two pieces during bend testing, the CPI-50 film still exhibited good flexibility.

Regarding the tensile tests, both CPI-20 and CPI-50 films showed measurable tensile strength after thermo-oxidative aging at 600 to 700 °C (Figure 5b); the specific data are displayed in Table S2. The unaged CPI-20 and CPI-50 films have a tensile strength of 161 and 108 MPa, respectively. After thermo-oxidative aging at 600 °C/30 min, the tensile strength reduced to 88 MPa for CPI-20 and 79 MPa for CPI-50. When the aging time extended to 60 min at 600 °C, the tensile strength was reduced to 23 MPa (CPI-20) and 42 MPa (CPI-50), indicating that the thermo-oxidation at 600 °C increased with extending aging time. Moreover, after aging at 700 °C for 5, 15, and 30 min, the CPI-50 film still kept a tensile strength of 63, 35, and 18 MPa, successfully, and its strength retention was much higher than that of CPI-20 (Figure 5c). Compared with boron-free films, the incorporation of a carborane cage into the polyimide backbone structure significantly improved the polymer thermo-oxidative stability at 700 °C.

### 3.4. Mechanism of Thermo-Oxidative Resistance

The extraordinary thermo-oxidative stabilities of the CPI films might be attributed to the formation of the boron oxide protective layer on the film surface, preventing the direct contact of inner polymer with oxygen molecules and resulting in the reduced decomposition rate of the inner polymer. Hence, the sheltered inner polymer could still provide the film with certain mechanical strength and flexibility even after thermo-oxidative aging at 700 °C. The thermal degradation kinetics in air was analyzed via TGA with the Flynn-Wall-Ozawa method [40,41], based on the following Equation (2), which is a derivate from Equation (1):

(1) $$\log\beta =\log\frac{AE_{a}}{Rg\left(\alpha \right)}-2.315-\frac{0.457E_{a}}{RT},$$

(2) $$\frac{d\log\beta }{d(1/T)}=\frac{0.457E_{a}}{R},$$

where *β* is the heating rate (K min^−1^), *R* is the gas constant (8.314 J moL^−1^ K^−1^), *E_a_* is the activation energy (kJ moL^−1^), *T* is the absolute temperature (K), and *g(α)* is the function of conversion rate *α*.

**Figure 6:**
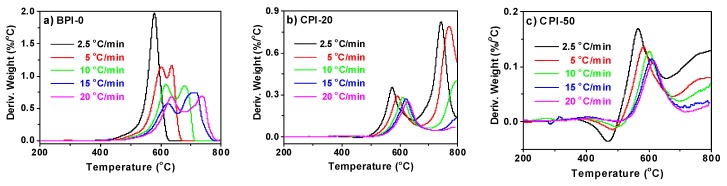
DTG curves of (**a**) CPI-0, (**b**) CPI-20, and (**c**) CPI-50 under various heating rates.

**Figure 7:**
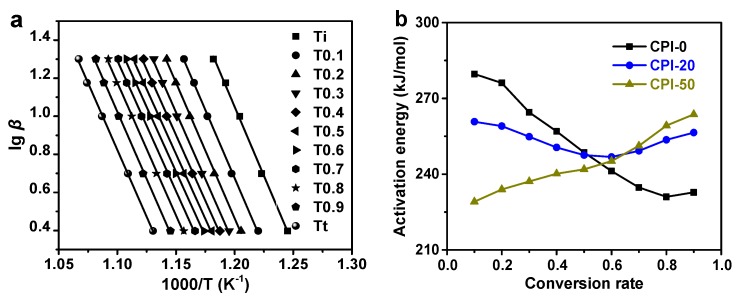
(**a**) Flynn-Wall-Ozawa plots for CPI-20 and (**b**) activation energy against conversion rate in the CPI films.

TGA of the CPI films with various boron-loadings (CPI-0, CPI-20, CPI-50) were performed in air at different heating rates. Figure 6 compares the DTG (Derivative Thermogravimetry) curves of CPI films with different carborane loadings. The two intersection points of the two tangents of the decomposition peak and baseline were set as initial degradation temperature (*T_i_*) and terminal degradation temperature (*T_t_*). The initial weight residue was regarded as the weight residue at the initial degradation temperature and the same as the terminal weight residue. Then, the section between the initial and terminal weight residue was equally divided into 10 pieces, and each weight residue represented a certain conversion rate *α*. According to the conversion rate *α* and heating rate *β*, *T_α_* (temperature at conversion rate *α*) could be directly determined in the corresponding TGA curves. Subsequently, plots of lg *β* against 1000/*T* were linearly fitted to a series of straight lines in which the slopes were used to compute the activation energy *E_a_* at the corresponding conversion rate *α*, as exhibited in Figure 7a. All of the generated lines were in excellent linear dependence with R^2^ > 0.98, as summarized in Table S3.

Obviously, the incorporation of carborane lowered the initial activation energy of the polyimide system (Figure 7b). Since the degradation activation energy *E_a_* was defined as the energy barrier that should be overcome to activate the thermal decomposition, the introduction of a flexible, bulky carborane cage loosened the chain packing, resulting in the lower initial *E_a_*. With an increase in oxidation time, the *E_a_* of CPI-0 diminished drastically while that of CPI-50 ascended gradually, probably due to the formation of a boron oxide protective layer during the decomposition. The formed boron oxide layer covered on the film surface could shelter the inner polymer, thus enhancing the energy barrier. In addition, the increase in the energy barrier, in turn, slowed down the decomposition process of the inner polymer, and the degradation rate decreased little by little as decomposition proceeded. At the conversion rate *α* > 0.5, the *E_a_* of CPI-50 exceeded that of CPI-0, and it continued to grow while the *E_a_* of CPI-0 continued to diminish (Figure 7b). Furthermore, the *E_a_* of CPI-20 film declined at first and then rose up later, implying that the boron content was too low to yield a sufficient protective layer to enhance the *E_a_* at the beginning. Compared with CPI-0, the protective layer only retarded the degradation rate of CPI-20, but when the conversion rate α > 0.6, the layer was dense enough to protect the inner polymer, leading to the growth of *E_a_* in which the value was between CPI-0 and CPI-50.

**Figure 8:**
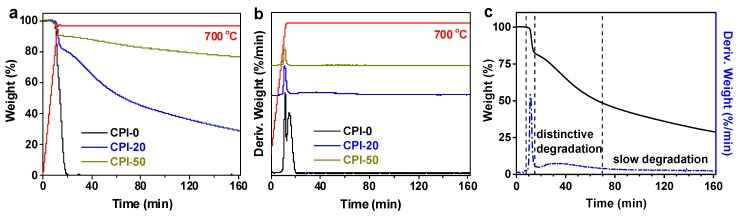
(**a**) TGA and (**b**) DTG curves of CPI-0, CPI-20, and CPI-50 isothermally treated at 700 °C for 150 min in air, and (**c**) three degradation stages of CPI-20 film.

To further investigate the mechanism, three representatives with different carbon-loadings (CPI-0, CPI-20, and CPI-50) were isothermally aged by TGA in air at 700 °C for 150 min (Figure 8a). Apparently, the thermo-oxidative stability was greatly improved by increasing the carborane loadings. Since the boron-free CPI film (CPI-0) was completely decomposed into volatiles rapidly, whereas the CPI-20 and CPI-50 film still maintained more than 29% and 76% weight residue after 150 min of thermo-oxidative degradation at 700 °C, respectively. In addition, the boron-free CPI film (CPI-0) was easier to decompose with a degradation peak more intense than the carborane-containing films (CPI-20 and CPI-50). Combined with DTG curves (Figure 8b), a second sharp weight loss peak immediately followed the first for CPI-0 in air. However, only one decomposition peak was observed for CPI-50 in air. This could be interpreted by the fact that the further degradation of the CPI-50 films was postponed due to the higher *E_a_* of the boron oxide passivation layer generated during the thermal treatment. It was noteworthy that a new broad peak was generated between the sharp decomposition peak and the slow degradation region for CPI-20 in the DTG curves (Figure 8c), manifesting that there was a distinctive degradation from 5 to 60 min in isothermal treatment.

**Figure 9:**
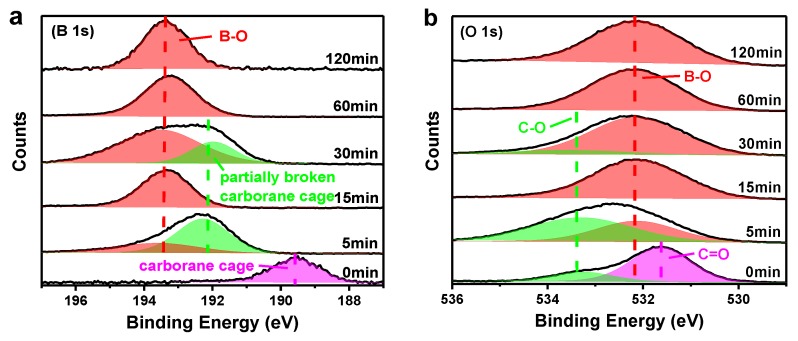
(**a**) B 1s and (**b**) O 1s XPS spectra of CPI-20 isothermally treated at 700 °C.

XPS was measured to investigate the chemical changes in the binding energies of boron and oxygen elements of the CPI films thermally treated at different conditions (Figure 9). The test samples of the CPI-20 film were cleaned by deionized water and ethanol for several times to wash out the impurities on the surfaces and then heated to 700 °C in a muffle furnace standing for various times (5 min, 15 min, 30 min, 60 min, and 120 min). After thermo-oxidative aging for 5 min, the polyimide located on the out of surface was initially decomposed, evidenced by the disappearance of the carborane cage peaks (189.6 eV) and C=O bonds (531.6 eV), respectively, and the absorption intensities of C-O (533.3 eV) in the O 1s spectrum became more conspicuous. Furthermore, the signal that occurred around 192.2 eV was assigned to the partially destroyed carborane cage, owing to the pyrolysis [42]. Meanwhile, the boron atoms linked in the carborane cage were reacted with oxygen to form a boron oxide layer on the surface evidenced by the appearance of B-O bonds in B 1s (193.4 eV) and O 1s (532.2 eV) spectra in the initial 5 min. Then, a dense passivation layer was covered on the surface, because only the B-O peak was observed both in B 1s and O 1s spectra (15 min). Nevertheless, when the aging time extended to 30 min, the partially broken carborane cage (192.3 eV) and C-O (533.3 eV) signals were reappeared in B 1s and O 1s spectra, respectively, which was in well accordance with the fact that the degradation rate of CPI-20 during 5 to 60 min was faster than that of CPI-50 while slower than CPI-0′s. It was presumed that, during 5 to 60 min, there was a dynamic equilibrium in the breakage and regeneration of the passivation layer, as a result of the disappearance and reappearance of partially damaged carborane cage and C-O peaks. With the aging time increased, the boron oxide layer became increasingly denser, evidenced by the fact that only the B-O peak was collected.

**Figure 10:**
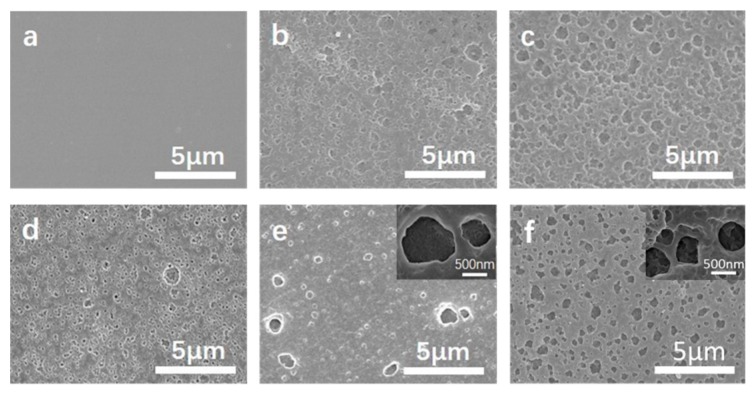
SEM images of the surfaces of CPI-20 samples heated at 700 °C in air for (**a**) 0 min; (**b**) 5 min; (**c**) 15 min; (**d**) 30 min; (**e**) 60 min, and (**f**) 120 min.

**Figure 11:**
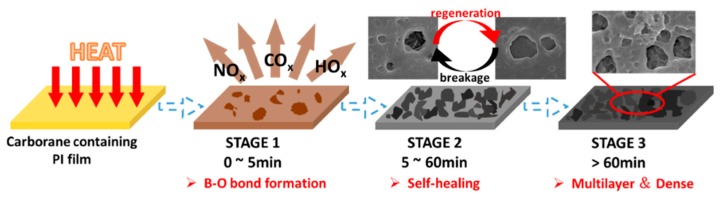
Mechanism of the three stages during thermal degradation.

Figure 10 compares the surface morphologies of CPI-20 samples after thermo-oxidative aging at 700 °C for different times. The thermal degradation could be divided into three stages, as shown in Figure 11. Based on the XPS analysis, the first stage was the decomposition of the external polymer, and the surface was gradually rougher. In this stage, the outmost film was reacted with oxygen, and the volatiles, such as oxynitride, oxycarbide, and hydroxide, were eliminated, accompanied by the formation of a B-O bond. The second stage was the generation of the boron oxide protective layer on the film surface. Initially, part of the surface was covered by that oxidative layer, as shown in Figure 9 (5 min) and Figure 10b. Then, the polymer next to the opened hole on the outmost passivation layer was degraded and formed a new passivation layer in situ, which enabled the hybrid polyimide surface to be “self-healing” [43], as shown in Figure 9. In this stage, breakage and regeneration of the passivation layer were in a dynamic equilibrium, but the regenerated layers gradually repaired the breakage of the previous one, ultimately forming the multilayered protective layer (Figure 10e). This layer covered the film surface and prevented the direct contact of the inner polymer and oxygen, thus significantly reducing the degradation rate. Although a “self-healing”, multilayered, dense passivation layer was generated on the film surface during the second stage, the heat could still be transferred into the interior via the protective layer, so the polymer was slowly degraded in the last stage.

## 4. Conclusions

Carborane-containing polyimide (CPI) films with ultrahigh thermal and thermo-oxidative stability up to 700 °C were successfully synthesized. The excellent thermal and thermo-oxidative stability was attributed to the multilayered protection layers formed on the film surface by thermal conversion of the carborane group into boron oxides. The boron oxide layer enhanced the degradation activation energy and suppressed the direct contact of inner polymer materials with oxygen molecules in a high-temperature environment, acting as a “self-healing” skin layer on the polyimide materials. Compared with the corresponding carborane-free films, the CPI films retained much higher mechanical strength and flexibility even after thermo-oxidative aging at 700 °C/30 min. It was this “self-healing”, multilayered, dense boron oxide protective layer that slowed down the further erosion process of the inner polymer and prolonged the service life of materials at high temperatures.

## Figures and Tables

**Table 1:** Calculated and experimental values of each element in the PAA resin (PAA-50).

Elemental Contents	C (%)	N (%)	H (%)	O (%)	B (%)
Calculated values	61.51	5.33	4.14	18.73	10.29
Elemental analysis	62.01	5.44	4.02	-	-
XPS analysis	60.30	4.94	-	19.55	11.07

-: The elemental analysis cannot detect the contents of B and O; XPS analysis can not detect the content of H, but the contents of other elements were in great agreement with the calculated values.

**Table 2:** Bending results of the CPI films after thermo-oxidative aging at various conditions.

Temperature/Time	600 °C/30 min	600 °C/60 min	700 °C/5 min	700 °C/15 min	700 °C/30 min
CPI-0	--	--	--	--	--
CPI-20	++	++	++	++	+
CPI-50	++	++	++	++	++

++: bendable; +: unbendable but can be conducted on tensile test; --: broken.

## Supplementary Materials

The following are available online at https://www.mdpi.com/2073-4360/11/12/1930/s1, Figure S1: The images of the carborane containing PAA solutions, Table S1: Thermal properties of the CPI films, Table S2: Tensile properties of CPI-20 and CPI-50 after thermo-oxidative aging, Table S3: Degradation *E_a_* and linearly dependent coefficients R^2^ of CPI-0, CPI-20 and CPI-50 and Scheme S1: Synthesis of 1,7-bis (aminophenyl)-*meta*-carborane (BACB).
