# Supplementary material for: Carborane-Containing Aromatic Polyimide Films with Ultrahigh Thermo-Oxidative Stability
Source: Polymers (Basel). 2019 Nov 22;11(12):1930. doi: 10.3390/polym11121930 (PMC6960542; doi:10.3390/polym11121930)
Supplement: Supplementary file 1 [file polymers-11-01930-s001.pdf]

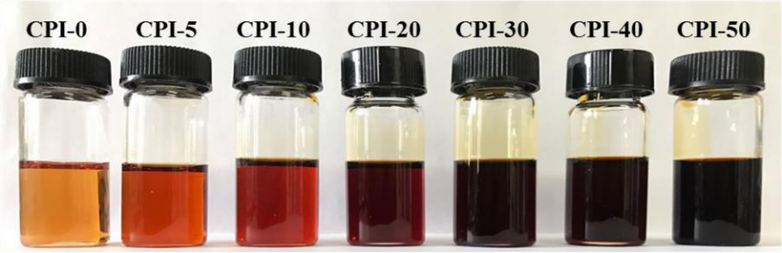

Figure S1. The images of the carborane containing PAA solutions.

Table S1. Thermal properties of the CPI films.

CPIs	T_g [a] [°C]	T_5 [b] [℃]		T_{10} [c] [°C]		R_w [d] [%]	
		N_2	Air	N_2	Air	N_2	Air
CPI-0	304	582	574	605	598	68.6	5.3
CPI-5	320	601	602	622	623	71.2	64.8
CPI-10	329	598	604	623	626	74.4	69.0
CPI-20	340	597	606	627	628	79.4	76.2
CPI-30	352	596	611	630	632	82.6	81.2
CPI-40	366	595	615	642	653	85.9	86.5
CPI-50	371	596	632	657	749	87.9	90.0

[a] T_g: tan delta peak value determined by DMA; [b] T_5: decomposition temperature at 5% weight loss; [c] T_{10}: decomposition temperature at 10% weight loss; [d] R_w: the char yield at 750 °C.

Table S2. Tensile properties of CPI-20 and CPI-50 after thermo-oxidative aging.

Aging Condition	CPI-20		CPI-50	
	T_M [a] [GPa]	T_S [b] [MPa]	T_M [GPa]	T_S [MPa]
initial	4.9	161	3.7	108
600 °C/30 min	3.0	88	2.9	79
600 °C/60 min	- [c]	23	-	42
700 °C/ 5 min	1.8	45	2.5	63
700 °C/15 min	-	21	1.7	35
700 °C/30 min	-	7	-	18

[a] T_M: tensile modulus; [b] T_S: tensile strength; [c] -: can not be evaluated.

Table S3. Degradation E_a and linearly dependent coefficients R^2 of CPI-0, CPI-20 and CPI-50.

α	CPI-0		CPI-20		CPI-50	
	E_a [a] [kJ mol^{-1}]	R^2 [b]	E_a [kJ mol^{-1}]	R^2	E_a [kJ mol^{-1}]	R^2
0	278.7	0.993	264.1	0.992	222.8	0.981
0.1	279.7	0.993	260.8	0.998	229.1	0.989
0.2	276.1	0.990	259.0	0.994	234.0	0.991
0.3	264.4	0.986	254.8	0.990	237.1	0.992
0.4	257.0	0.988	250.6	0.998	240.2	0.992
0.5	248.6	0.990	247.5	0.989	241.9	0.992
0.6	241.2	0.993	246.8	0.997	245.2	0.991

0.7	234.7	0.994	249.2	0.991	251.2	0.992
0.8	231.0	0.997	253.6	0.990	259.2	0.997
0.9	232.8	0.998	256.5	0.997	263.7	0.998
1	230.0	0.992	255.6	0.988	274.0	0.992

[a] E_a: degradation activation energy computed via Equation 2; [b] R^2: linear dependence of the lines fitted with plots of lg β against 1000/T.

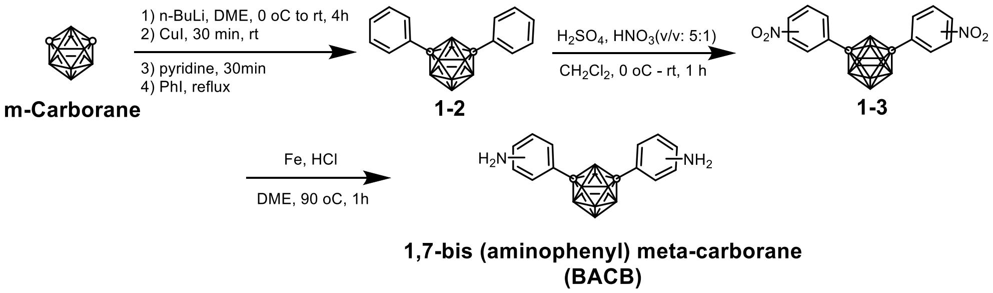

Scheme S1. Synthesis of 1,7-bis (aminophenyl)-*meta*-carborane (BACB).

There were three steps to synthesize the 1,7-bis (aminophenyl)-*meta*-carborane (BACB), and the yields were 81%, 91% and 82%, respectively. Ultimately, faint yellow solid of BACB was obtained and the characterization was shown as follow:

^{1}H NMR (300 MHz, $CDCl_3$): δ = 1.1 – 4.5 (br, 10H), 3.61 (s, 4H), 6.48 – 6.59 (m, 2H), 6.75 – 6.86 (m, 3H), 6.98 – 7.23 (m, 3H) ppm. ^{13}C NMR (300 MHz, $CDCl_3$): δ = 77.20, 77.93, 78.28, 114.32, 114.54, 115.16, 118.05, 125.24, 125.36, 128.76, 129.17, 136.33, 146.19, 146.71, 146.76 ppm. ^{11}B NMR (500 MHz, $CDCl_3$): δ = -6.08, -10.21, -10.87, -11.94, -13.10 ppm. HRMS (ESI): m/z calcd for $C_{14}H_{22}B_{10}N_2[M]^+$:327.2750. Found: 327.2872.

© 2019 by the authors. Submitted for possible open access publication under the terms and conditions of the Creative Commons Attribution (CC BY) license (http://creativecommons.org/licenses/by/4.0/).
